# A BioBrick compatible strategy for genetic modification of plants

**DOI:** 10.1186/1754-1611-6-8

**Published:** 2012-06-20

**Authors:** Patrick M Boyle, Devin R Burrill, Mara C Inniss, Christina M Agapakis, Aaron Deardon, Jonathan G DeWerd, Michael A Gedeon, Jacqueline Y Quinn, Morgan L Paull, Anugraha M Raman, Mark R Theilmann, Lu Wang, Julia C Winn, Oliver Medvedik, Kurt Schellenberg, Karmella A Haynes, Alain Viel, Tamara J Brenner, George M Church, Jagesh V Shah, Pamela A Silver

**Affiliations:** 1 Department of Systems Biology, Harvard Medical School, Boston, MA, 02115, USA; 2 Harvard College, Harvard University, Cambridge, MA, 02138, USA; 3 Department of Molecular and Cellular Biology, Harvard University, Cambridge, MA, 02138, USA; 4 The Arnold Arboretum of Harvard University, Boston, MA, 02131, USA; 5 Wyss Institute for Biologically Inspired Engineering, Harvard University, Boston, MA, 02115, USA; 6 Department of Genetics, Harvard Medical School, Boston, MA, 02115, USA; 7 Current Address: Department of Chemical and Biomolecular Engineering, University of California, Los Angeles, CA, 90095, USA; 8 Current Address: School of Biological and Health Systems Engineering, Arizona State University, Tempe, AZ, 85287, USA

**Keywords:** iGEM, Synthetic biology, Arabidopsis, Plant biotechnology

## Abstract

**Background:**

Plant biotechnology can be leveraged to produce food, fuel, medicine, and materials. Standardized methods advocated by the synthetic biology community can accelerate the plant design cycle, ultimately making plant engineering more widely accessible to bioengineers who can contribute diverse creative input to the design process.

**Results:**

This paper presents work done largely by undergraduate students participating in the 2010 International Genetically Engineered Machines (iGEM) competition. Described here is a framework for engineering the model plant *Arabidopsis thaliana* with standardized, BioBrick compatible vectors and parts available through the Registry of Standard Biological Parts (http://www.partsregistry.org). This system was used to engineer a proof-of-concept plant that exogenously expresses the taste-inverting protein miraculin.

**Conclusions:**

Our work is intended to encourage future iGEM teams and other synthetic biologists to use plants as a genetic chassis. Our workflow simplifies the use of standardized parts in plant systems, allowing the construction and expression of heterologous genes in plants within the timeframe allotted for typical iGEM projects.

## Background

Selective breeding has long been used to modify plant characteristics such as growth rate, seed size, and flavor [1]. For much of agricultural history, the targeted traits reflected the needs of local growers and consumers, creating a vast array of crop varieties. Advances in the field of genetics and the advent of recombinant DNA technology accelerated our ability to manipulate food crops [1-5]. In particular, the introduction of multiple genes (termed *gene stacking* in plants) has made plants accessible to synthetic biology applications [6-11]. In contrast to previous developments in agricultural technology, genetic modification of plants has been primarily performed for the benefit of large-scale monocultures of agricultural crops.

**Figure 1:**
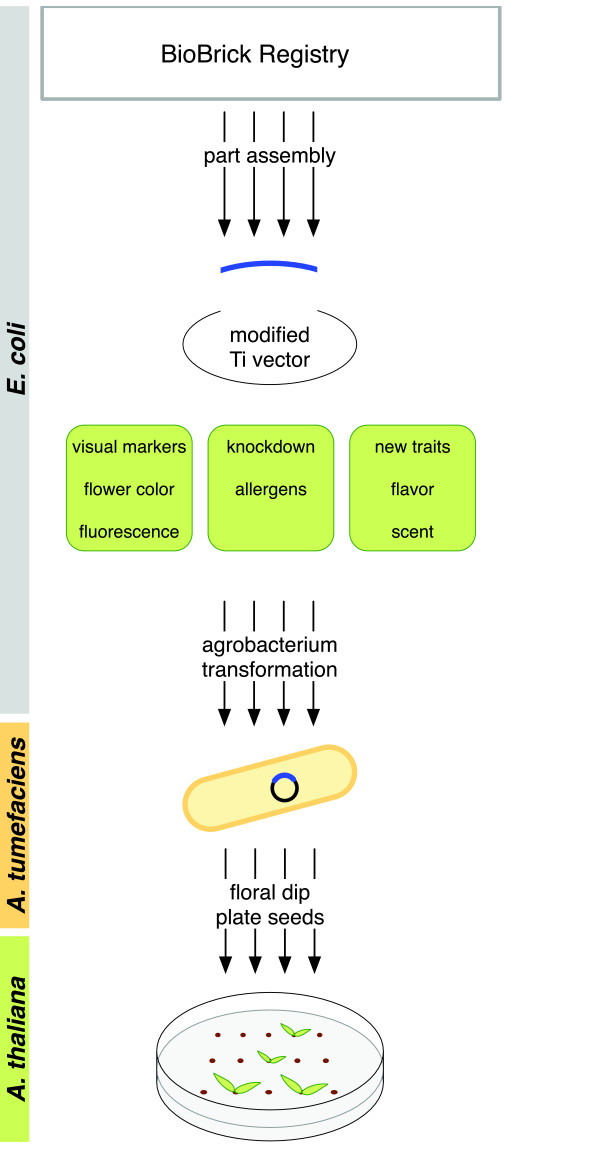
**A standardized, modular system for the production of genetically-modified plants.** Genetic parts (such as those obtained from the BioBrick Registry) were assembled and inserted into modified vectors (Open, Expression, or Reporter) in *E. coli*. These parts may be assembled to build constructs to impact a wide variety of plant phenotypes. Once assembled, these vectors were transformed into *Agrobacterium*. Using the floral dip procedure, *Agrobacterium* infected Arabidopsis, thereby transferring the assembled construct. Once seeds were produced, they were plated on selective media to obtain transgenic plants carrying the assembled construct.

This work aims to create a standardized, modular system for the production of genetically enhanced plants to facilitate their adoption by diverse users. Ideally, a plant engineering system is customizable, yet has convenient standard features that minimize the need to re-invent common steps such as transferring genetic material into the plant. We demonstrate the feasibility of small-scale engineering projects in the model organism, *Arabidopsis thaliana* (Arabidopsis), using a BioBrick-modified plant vector system (Figure 1), performed within the time constraints of the iGEM competition.

Using BioBrick compatible plant vectors, we sought to modify the taste of Arabidopsis, specifically enhancing the sweetness of a bitter plant without altering sugar content. Several naturally occurring proteins are 100–3000 times sweeter than sugar by weight [12]. Brazzein, monellin, thaumatin, pentadin, mabinlin, and curculin are sweet proteins found in a variety of African and South Asian fruits, with no sequence similarity or common features [13]. Brazzein, isolated from the West African fruit *Pentadiplandra brazzeana*, is the smallest of these proteins with only 54 amino acids. It is exceptionally heat stable and was previously expressed heterologously in *Escherichia coli* (*E. coli*) [13], *Zea mays *[14], and *Lactobacillus lactis *[15].

Miraculin, isolated from the berries of the West African plant *Synsepalum dulcificum*, does not taste sweet on its own. Rather, it acts as a flavor-inverter by binding to taste receptors on the tongue in a pH-dependent manner, causing sour foods to taste sweet [16]. A 1 μM miraculin solution is sufficient to activate this inversion, where 20 mM citrate corresponds to the sweetness of 300 mM sucrose [17]. Miraculin is a glycosylated homodimer that has been heterologously expressed in lettuce [18], tomato [19], and even *E. coli *[17], indicating that endogenous *S. dulcificum* glycosylation is not required for functional expression.

Beyond creative gastronomy, we imagine this system being used to enhance the nutritional content of edible plants or help allergy sufferers enjoy the benefits of fresh home-grown produce (Figure 1). The development of efficient transformation techniques for many plants remains a key hurdle for commercial and personal agriculture. However, flexible genetic customization of plants also requires a system of easily transferable, standardized components such as those presented here. We hope this work will lead to techniques that yield a diversity of produce tailored to individual, community, and local environmental needs.

## Results

### Design of BioBrick compatible vectors for Arabidopsis transformation

Arabidopsis is readily transformed by *Agrobacterium*: when a plant is injured, *Agrobacterium* migrates to the wound site and transfers the T-DNA region of its tumor-inducing (Ti) plasmid into the plant cell [20]. The T-DNA localizes to the nucleus and integrates into the plant’s chromosomal DNA. A series of vectors (the pORE series) have been developed from *Agrobacterium*’s Ti plasmid to allow transformation of heterologous DNA into plants via *Agrobacterium *[20]. pORE vectors come equipped with a multiple cloning site (MCS) containing twenty-one unique restriction endonuclease sites. Reporters or promoters are included to create expression vectors, reporter vectors, or vectors that can carry an exogenous promoter or open reading frame. This vector series offers either glufosinate resistance via the *pat* gene, or kanamycin resistance via the *nptII* gene, to enable the selection of successfully transformed plants.

**Figure 2:**
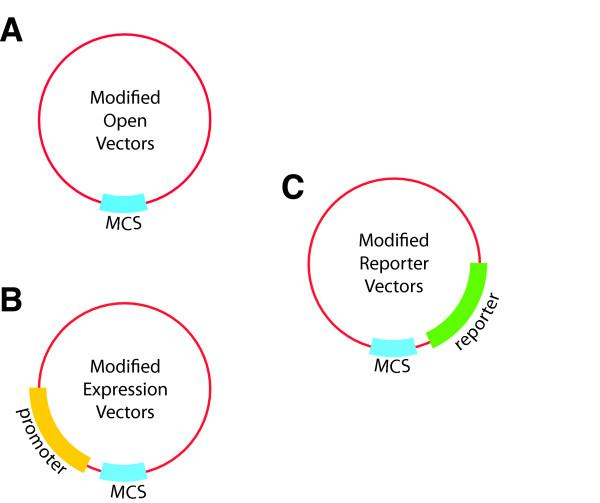
**Schematic of BioBrick plant vectors. (A)** Modified Open vectors are based on vectors pORE O1 and O2 [14]. They are designed for general insertion of a construct. **(B)** Modified Expression vectors are based on vectors pORE E3 and E4 [14]. They contain an inducible promoter preceding the BioBrick MCS, to permit user-controlled expression of the inserted construct. **(C)** Modified Reporter vectors are based on vectors pORE R1 and R2 [14]. They contain a reporter gene following the BioBrick MCS, such that expression of the reporter follows that of the inserted construct.

We developed a new set of six BioBrick DNA assembly compatible plant transformation vectors based on the pORE series (Table 1). Vectors V1 and V2 (modified Open vectors) contain no promoter or reporter gene, allowing integration of constructs under the control of a chosen promoter (Figure 2A, Table 1). Vectors V3 and V4 (modified Expression vectors) contain the constitutive pENTCUP2 promoter upstream of the MCS (Figure 2B, Table 1), while V5 and V6 (modified Reporter vectors) contain no promoter but have either the reporter gusA or soluble modified GFP (smGFP) downstream of the cloning site (Figure 2C, Table 1). Each vector contains an MCS that is compatible with three widely used BioBrick standards (RFC 10, 20, 23, http://www.partsregistry.org).

**Table 1:** Features of BioBrick plant vectors

**Vector**	**BioBrick Registry ID**	**Bacterial Resistance**	**Plant Resistance**	**Promoter**	**Reporter**	**Original pORE vector**
V1	BBa_K382000	kan	pat	none	none	pORE O1
V2	BBa_K382001	kan	nptII	none	none	pORE O2
V3	BBa_K382002	kan	pat	pENTCUP2	none	pORE E3
V4	BBa_K382003	kan	nptII	pENTCUP2	none	pORE E4
V5	BBa_K382004	kan	nptII	none	gusA	pORE R1
V6	BBa_K382005	kan	nptII	none	smGFP	pORE R3

### Expression of standardized flavor protein genes in industrial microorganisms

**Figure 3:**
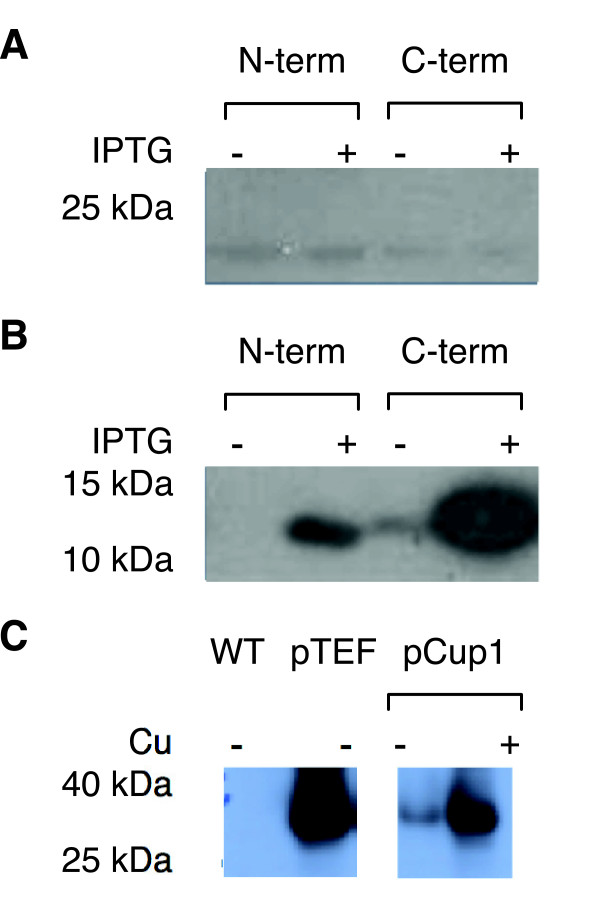
**BioBrick miraculin and brazzein protein expression in bacteria and yeast. (A)** Miraculin and **(B)** brazzein BioBricks were expressed from an IPTG-inducible promoter in *E. coli* with an N- or C-terminal Strep-II tag. Miraculin was expressed at low levels, with only a faint band appearing at 24 kDa. Brazzein was well expressed in an IPTG-dependent manner. **(C)** Brazzein BioBrick was expressed in yeast from pTEF or pCup1 promoters with a C- terminal Strep-II tag. Brazzein appeared larger in yeast vs. *E. coli*, most likely due to glycosylation of brazzein in yeast.

We first tested the expression of standardized miraculin and brazzein genes in *E. coli* and the yeast *Saccharomyces cerevisiae* (*S. cerevisiae*), since the introduction of exogenous genes is faster in these organisms. Full-length miraculin and brazzein genes were commercially synthesized and codon-optimized for expression in Arabidopsis. BioBrick compatible restriction enzyme sites bracketed each open reading frame. Constructs were tagged at either the N- or C-terminus with the Strep-II tag [21] for western blot analysis. Miraculin (Figure 3A) and brazzein (Figure 3B) were expressed from an IPTG-inducible T7 promoter in *E. coli*. Monomeric miraculin was expressed at very low levels at approximately 24 kDa regardless of tag location, which is consistent with previous work [17]. Brazzein was highly expressed in the same system at about 12 kDa, regardless of tag location, as has been previously observed [13]. Brazzein was also highly expressed from the constitutive TEF and copper-inducible CUP1 promoters in *S. cerevisiae* (Figure 3C). The higher molecular weight of the Strep-II tagged brazzein observed by western blot in yeast, compared to *E. coli* (~35 kDa versus 12 kDa) is likely due to yeast-specific glycosylation of the brazzein protein [22]. While expression of the miraculin gene was not verified in yeast, integration of both miraculin and brazzein constructs in Arabidopsis was attempted.

### Expression of flavor proteins in Arabidopsis

**Figure 4:**
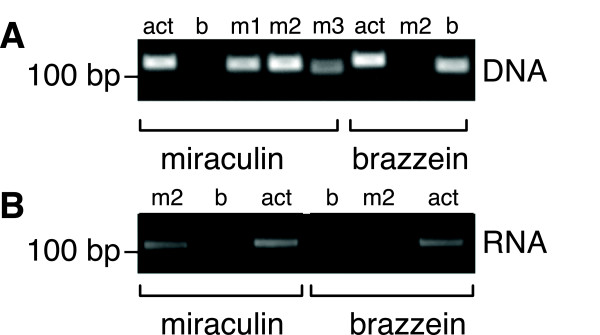
**BioBrick miraculin DNA and RNA expression in Arabidopsis. (A)** Integration of the miraculin and brazzein genes in the Arabidopsis genome was confirmed. Primer sets for miraculin (m) and brazzein (b) demonstrated that only the desired gene was integrated. **(B)** Miraculin mRNA was constitutively expressed in Arabidopsis however brazzein expression was not detected. act: actin control; b: brazzein primer set; m1-m3: miraculin primer sets.

We successfully introduced two different BioBrick plant vectors into Arabidopsis and selected for seeds carrying genomically-integrated miraculin and brazzein transgenes. Miraculin- or brazzein-encoding DNA under control of the pENTCUP2 promoter and NosT transcriptional terminator on either the V3 (glufosinate resistance) or V4 (kanamycin resistance) BioBrick vector was introduced into Arabidopsis via *Agrobacterium*-mediated transformation [23]. Transformed seeds were selected on MS-agar, and resistant plants were moved to soil and allowed to produce seeds. T1 generation seeds were collected and re-plated on selective plates. Resistant plants were once again moved to soil and allowed to produce T2 generation seeds. While integration of both the miraculin and brazzein genes into the plant genome was verified by PCR (Figure 4A), only miraculin RNA expression was detected by end-point PCR (Figure 4B). Miraculin expression could not be verified by western blot as the antibody showed significant background binding. However, the RNA expression data indicates that miraculin mRNA is expressed in our transgenic plants.

## Discussion and conclusions

Genetic engineering of plants at the industrial and laboratory scale is well established. Technological advances have yielded crops that reduce food production costs through resistance to pests, herbicide, drought, and flood [24]. Additionally, modification of crops (e.g., rice) to contain pro-vitamins can help treat health issues such as vitamin A deficiency in countries where staple foods do not provide the necessary nutrients [24,25]. However, advances in small-scale experimental horticulture, farming, and gardening have been impeded by the lack of readily available modular parts for the genetic modification of plants. Access to standardized plant vectors in the Registry of Standard Biological Parts will facilitate the design of small-scale plant engineering projects.

We have modified existing plant integration vectors to make them compatible with the BioBrick assembly standard 23 [26], demonstrated that they can be used to integrate transgenes in Arabidopsis, and showed successful integration and expression of the taste modifying gene miraculin. All constructs have been submitted to the Registry of Biological Parts (http://www.partsregistry.org) and are available as a resource for the synthetic biology and plant engineering communities. These include vectors modified from the pORE vector series [20], plant-specific regulatory elements (e.g., promoters, terminators), resistance markers, and the coding sequence of miraculin. The vector series features variations containing the constitutive promoter pENTCUP2 (V3 and V4), visible reporters gusA or smGFP (V5 and V6), or a simple multiple cloning site (V1 and V2), allowing expression of a gene from a promoter of choice.

In addition to using these vectors to express exogenous proteins, we have considered integrating constructs expressing hairpin RNAs [27] or artificial microRNAs [28] to knock down the expression of endogenous genes. This strategy is particularly powerful in that synthesizing a DNA sequence to match any gene transcript of choice allows the regulation of potentially any plant protein. For instance, this approach could be used reduce allergenic protein levels [29]. Alternatively, microRNAs could be targeted to metabolic regulators so that key metabolites, such as pigments or nutrients, are allowed to accumulate [30] and enhance the color or nutritional content of the plant. Modification of existing vectors to conform to a BioBrick assembly standard allows them to be integrated into a BioBrick cloning based workflow. In addition to simplifying the construction of more complex genetic devices, adhering to an assembly standard allows for the possibility of automation of the assembly process.

We hope that availability of plant integration vectors compatible with a common assembly standard will facilitate the use of plants as a chassis in synthetic biology. Local-scale design of food plants, in which the grower selects traits desired in their community, can be made possible through the availability of standardized, modular genetic parts. Personalized engineering of plants to modify flavor, nutritional value, or allergenicity could create a new class of designed foods that are grown and consumed at a local scale. We encourage the iGEM community to continue to explore these concepts via plant engineering.

A significant barrier to the adoption of local-scale plant engineering is the uncertain regulatory landscape for the deployment of genetically modified organisms. This landscape has been defined by large-scale commercial agriculture. As the tools of synthetic biology become more accessible, efforts by small groups such as our own will continue to challenge existing regulatory frameworks. We hope that our concept of genetic engineering tools in the hands of local growers will spur discussion and debate on how to responsibly regulate the synthetic biology scenarios of the near future.

## Materials and methods

### Plasmids and cloning

Gene assembly was performed in *E. coli* DH5α using BioBrick assembly standard 23 [26], and all described parts were submitted to the BioBrick Registry. Arabidopsis pORE series vectors were provided by The Arabidopsis Information Resource (TAIR) and engineered to support BioBrick cloning through PCR-based methods (see Additional file 1: Table S1). pORE Open Series vectors O1 and O2 were digested with SpeI and SacII and ligated with an annealed oligonucleotide insert with NheI and SacII overhangs containing the BioBrick Multiple Cloning Site (MCS) to create vectors V1 and V2. pORE Expression Series vectors E3 and E4 were digested with HindIII and SpeI and ligated with an insert PCR-amplified from the expression vectors containing a HindIII site upstream of the pENTCUP2 promoter and the BioBrick MCS and an NheI site downstream to create vectors V3 and V4. pORE Reporter Series vectors R1, containing the gusA reporter, and R3, containing the smGFP reporter, were digested with HindIII and SpeI and ligated with inserts containing the reporter gene PCR-amplified with primers containing a HindIII site followed by the BioBrick MCS upstream and NheI downstream, yielding vectors V5 and V6.

Brazzein and miraculin were codon-optimized for expression in Arabidopsis, commercially synthesized (Mr. Gene, Regensburg, Germany), and assembled with the pENTCUP2 promoter and NosT transcriptional terminator. Completed constructs were subcloned from BioBrick assembly vector V0120 to BioBrick modified pORE vectors through digestion with EcoRI and PstI.

### Plant maintenance

Wild-type Col-0 *Arabidopsis thaliana* seeds were sterilized by washing with 70% ethanol, 0.1% Triton X-100, followed by two 95% ethanol washes and two sterile dH_2_O washes. Seeds were then plated on 1X Murashige & Skoog (MS) media with 0.7% agar supplemented with 150 uM carbenicillin and placed in the dark at 4°C for three days before moving to an incubator with 16 h illumination at 20°C and 8 h dark at 15°C per day to allow seeds to germinate. Once plants produced secondary leaves, they were moved to soil and allowed to mature and produce seeds. Seeds were collected and stored at 4°C.

### Plant transformation

*Agrobacterium*-mediated transformation was performed according to previously reported techniques [23]. Briefly, *Agrobacterium* was made electro-competent by washing in cold sterile water and resuspending in 10% glycerol. Vector DNA was dialyzed to remove excess salt, and electroporated into *Agrobacterium*. Kanamycin-resistant colonies were grown in YEB media, spread on YEB plates, and allowed to form a lawn. Lawns were scraped and suspended in a solution of 20% YEB, 4% sucrose (w/v), and 0.024% Silwet L-77 surfactant (Helena Chemical Company, Collierville, TN). Wild-type Col-0 Arabidopsis flowers were dipped in the *Agrobacterium* solution and allowed to grow and develop seed pods. Seeds were collected from mature plants and selected on 1x MS media with 0.7% agar supplemented with 5 mg/L glufosinate or 50 μg/ml kanamycin.

### *E. coli* and yeast protein expression

In BL21(DE3) *E. coli*, StrepII-tagged brazzein and miraculin were inserted at multiple cloning site 1 of a BioBrick-modified pET-duet vector [31]. Cells were grown to mid-log phase and induced with a final concentration of 1 mM IPTG. Protein expression was measured by western blot.

In PSY580a yeast *S. cerevisiae*, StrepII-tagged brazzein was cloned with the constitutive TEF promoter or the copper-inducible CUP1 promoter and integrated at the LEU2 locus. Transformants were grown in YEPD media with 0.3 mM CuSO_4_ to induce protein expression, which was measured by western blot.

### Verification of genomic transgenes

Genomic DNA was extracted from Arabidopsis using the DNEasy kit (Qiagen) and amplified by PCR (see Additional file 1: Table S2). Whole cell RNA was collected using the plant RNEasy kit (Qiagen). cDNA was synthesized with the SuperScript III First-Strand synthesis kit (Invitrogen). qPCR was performed with primer pairs (see Additional file 1: Table S2) amplifying 100 base pair amplicons within target genes to identify expression of heterologous genes or endogenous gene knockdown.

### SDS-page and western blotting

Protein samples were extracted from Arabidopsis, *E. coli*, and yeast and normalized using the Bradford assay (Bio-Rad, Hercules, CA). Samples were diluted into SDS-PAGE loading buffer and loaded onto a 4–20% Tris/glycine/SDS acrylamide gel. α-Strep-tag II antibody (HRP-conjugated, Novagen, Gibbstown, NJ) was used to measure brazzein and miraculin protein expression in *E. coli* and yeast, and α-miraculin antibody [18] (provided by Tadayoshi Hirai, Graduate School of Life and Environmental Sciences, University of Tsukuba, Japan) was used to detect levels of miraculin expression in Arabidopsis. Monoclonal Anti-β-Tubulin antibody (Sigma-Aldrich, St-Louis, MO) was used to detect tubulin in Arabidopsis.

## Abbreviations

MCS: Multiple cloning site.

## Competing interests

The authors declare they have no competing interests.

## Authors' contributions

Cloning schemes were designed by PMB, DRB, MCI, CMA, AD, JGdW, MAG, JYQ, MLP, AMR, MRT, LW, JCW, and OM. KS provided technical assistance with Arabidopsis culture and *Agrobacterium*-mediated transformation. PMB, DRB, MCI, and CMA performed PCR, qPCR and western blots; all other cloning and experiments were performed by AD, JGdW, MAG, JYQ, MLP, AMR, MRT, LW, and JCW. KAH, AV, TJB, GMC, JVS, and PAS provided general advising throughout the project. The manuscript was drafted by PMB, DRB, MCI, and CMA. All authors read and approved the final manuscript.

## Supplementary Material

Additional file 1 **Supplementary Data for A BioBrick Compatible Strategy for Genetic Modification of Plants.** Contains tables of primer sequences describing primers used to modify pORE vectors and verify integration and RNA expression of transgenes. Click here for file
