# Supplementary material for: A BioBrick compatible strategy for genetic modification of plants
Source: J Biol Eng. 2012 Jun 20;6:8. doi: 10.1186/1754-1611-6-8 (PMC3537565; doi:10.1186/1754-1611-6-8)
Supplement: Additional file 1 — Supplementary Data for A BioBrick Compatible Strategy for Genetic Modification of Plants. Contains tables of primer sequences describing primers used to modify pORE vectors and verify integration and RNA expression of transgenes. [file 1754-1611-6-8-S1.pdf]

Supplementary Data for A BioBrick Compatible Strategy for Genetic Modification of Plants.

Patrick M. Boyle[1*], Devin R. Burrill[1*], Mara C. Inniss[1*], Christina M. Agapakis[1‡*], Aaron Deardon[2†], Jonathan G. DeWerd[2†], Michael A. Gedeon[2†], Jacqueline Y. Quinn[2†], Morgan L. Paull[2†], Anugraha M. Raman[2†], Mark R. Theilmann[2†], Lu Wang[2†], Julia C. Winn[2†], Oliver Medvedik[3], Kurt Schellenberg[4], Karmella A. Haynes[1¶], Alain Viel[3], Tamara J. Brenner[3], George M. Church[5,6], Jagesh V. Shah[1§], and Pamela A. Silver[1,5§]

[1] Department of Systems Biology, Harvard Medical School, Boston, MA 02115
[2] Harvard College, Harvard University, Cambridge, MA 02138
[3] Department of Molecular and Cellular Biology, Harvard University, Cambridge, MA 02138
[4] The Arnold Arboretum of Harvard University, Jamaica Plain, MA 02131
[5] Wyss Institute for Biologically Inspired Engineering, Harvard University, Boston, MA 02115
[6] Department of Genetics, Harvard Medical School, Boston, MA 02115

[*] These authors contributed equally to this work
[†] These authors contributed equally to this work
[§] Corresponding authors
[‡] Current Address: Department of Chemical and Biomolecular Engineering, University of California, Los Angeles, CA 90095
[¶] Current Address: School of Biological and Health Systems Engineering, Arizona State University, Tempe, AZ 85287

Supplementary Tables

Supplmentary Table 1 - Primers used to engineer Arabidopsis pORE series transformation vectors (TAIR*).

Primer Name	Sequence	Purpose
O_5’	GGGAATTcgcggccgcttctagaactagtagcggccgctgcaga	Add MCS** to pORE Open Series vectors O1 and O2 to create V1 and V2
O_3’	CTAGTctgcagcggccgctactagttctagaagcggccgcgAATTCccgc	Add MCS** to pORE Open Series vectors O1 and O2 to create V1 and V2
E_5’	cgtgatAAGCTTgggatcttctgcaagcatctc	Add MCS** to pORE Expression Series vectors E3 and E4 to create V3 and V4
E_3’	caagaGCTAGCctgcagcggccgctactagtcctctaattctagaagcggccgcgaattctccggtgggttttgaggtgag	Add MCS** to pORE Expression Series vectors E3 and E4 to create V3 and V4
R1_5’	actacgaagcttgaattcgcggccgcttctagaattagaggactagtagcggccgctgcagAgagctcatgttacgtcctgtagaaacc	Add MCS** to pORE Reporter Series vector R1 to create V5
R1_3’	caagaGCTAGCAaaaggtacctcattgtttgc	Add MCS** to pORE Reporter Series vector R1 to create V5
R3_5’	actacgaagcttgaattcgcggccgcttctagaattagaggactagtagcggccgctgcagAgagctcatggcgagtaaaggagaagaac	Add MCS** to pORE Reporter Series vector R3 to create V6
R3_3’	atctGCTAGCttttggtaccttatttgtatag	Add MCS** to pORE Reporter Series vector R3 to create V6

*Original vectors were provided by The Arabidopsis Information Resource (TAIR)

**MCS: BioBrick multiple cloning site

Supplmentary Table 2 - Primers for testing DNA and RNA expression of miraculin and brazzein in Arabidopsis.

Primer Name	Sequence
BRAZ_5’	AATTGGCAAACCAGTGCAA
BRAZ_3’	TCACAGATACACTGGAGGTTCC
MIR1_5’	CGTGACTATCGGAGGAGTGAAGGGT
MIR1_3’	CCACACACAGTTGGGCAAAACACA
ACTIN_5’	TGTGCCAATCTACGAGGGTTT
ACTIN_3’	TTTCCCGCTCTGCTGTTGT
